# A review of visceral leishmaniasis during the conflict in South Sudan and the consequences for East African countries

**DOI:** 10.1186/s13071-016-1743-7

**Published:** 2016-08-22

**Authors:** Waleed Al-Salem, Jennifer R. Herricks, Peter J. Hotez

**Affiliations:** 1 Ministry of Health, Riyadh, Saudi Arabia; 2 Department of Pediatrics and Molecular Virology and Microbiology, National School of Tropical Medicine, Baylor College of Medicine, Houston, TX USA; 3 James A. Baker III Institute for Public Policy, Rice University, Houston, TX USA; 4 Sabin Vaccine Institute and Texas Children’s Hospital Center for Vaccine Development, Houston, TX USA; 5 Department of Biology, Baylor University, Waco, TX USA

**Keywords:** Visceral leishmaniasis, East Africa, South Sudan, Refugees, Civil war, Conflict zone, *Leishmania donovani*, *Phlebotomus orientalis*

## Abstract

**Background:**

Visceral leishmaniasis (VL), caused predominantly by *Leishmania donovani* and transmitted by both *Phlebotomus orientalis* and *Phlebotomus martini,* is highly endemic in East Africa where approximately 30 thousands VL cases are reported annually. The largest numbers of cases are found in Sudan - where *Phlebotomus orientalis* proliferate in *Acacia* forests especially on Sudan’s eastern border with Ethiopia, followed by South Sudan, Ethiopia, Somalia, Kenya and Uganda. Long-standing civil war and unrest is a dominant determinant of VL in East African countries. Here we attempt to identify the correlation between VL epidemics and civil unrest.

**Objective and methodology:**

In this review, literature published between 1955 and 2016 have been gathered from MSF, UNICEF, OCHA, UNHCR, PubMed and Google Scholar to analyse the correlation between conflict and human suffering from VL, which is especially apparent in South Sudan.

**Findings:**

Waves of forced migration as a consequence of civil wars between 1983 and 2005 have resulted in massive and lethal epidemics in southern Sudan. Following a comprehensive peace agreement, but especially with increased allocation of resources for disease treatment and prevention in 2011, cases of VL declined reaching the lowest levels after South Sudan declared independence. However, in the latest epidemic that began in 2014 after the onset of a civil war in South Sudan, more than 1.5 million displaced refugees have migrated internally to states highly endemic for VL, while 800,000 have fled to neighboring countries.

**Conclusion:**

We find a strong relationship between civil unrest and VL epidemics which tend to occur among immunologically naïve migrants entering VL-endemic areas and when *Leishmania*-infected individuals migrate to new areas and establish additional foci of disease. Further complicating factors in East Africa’s VL epidemics include severe lack of access to diagnosis and treatment, HIV/AIDS co-infection, food insecurity and malnutrition. Moreover, cases of post-kala-azar dermal leishmaniasis (PKDL) can serve as important reservoirs of anthroponotic *Leishmania* parasites.

## Background

Visceral leishmaniasis (VL), also known as kala-azar, is a serious and often fatal neglected tropical disease (NTD) that is highly correlated with war, poverty and failed health systems [1, 2]. The latest estimates from the Global Burden of Disease Study 2013 indicate that VL resulted in 62,500 deaths in the year 2013, following only malaria as the leading cause of death from parasitic infections [3]. It is further estimated that up to 389,100 cases occur annually, causing 4.24 million disability-adjusted life years (DALYs) such that VL is the highest-ranking NTD in terms of disease burden [4, 5]. Beyond this high level of global morbidity is a huge economic impact, as well as social stigmatization and other psychological consequences [6–8]. By some estimates over 90 % of VL cases are reported in six countries, namely Bangladesh, Brazil, Ethiopia, India, South Sudan and Sudan [9].

The East African countries of Sudan, South Sudan, Ethiopia, Kenya, Uganda and Somalia compose one of the main geographic areas hardest hit by VL, where it is mostly caused by *Leishmania donovani*. Phlebotomine sand flies are widely distributed across East Africa and various subgenera have been reported including *Phlebotomus*, *Paraphlebotomus*, *Synphlebotomus*, *Larroussius* and *Anaphlebotomus* [10]. Suspected vectors of *L. donovani* in East Africa include *Phlebotomus* (*Larroussius*) *orientalis*, *Phlebotomus* (*Synphlebotomus*) *martini*, *Phlebotomus* (*Anaphlebotomus*) *rodhaini* and *Phlebotomus* (*Synphlebotomus*) *celiae* [11, 12]. Furthermore, *Leishmania donovani* has been reported as responsible for both cutaneous leishmaniasis and mucosal leishmaniasis in East Africa [13–15].

According to Alvar et al. [5] the annual incidence of VL in East Africa is between 29,400 and 56,700 cases, accounting for approximately 15 % of the global cases. However, these numbers can vary considerably from year to year due to ongoing political and climatic problems in East African countries, including civil wars, social unrest, forced displacements, migration, unusual rainfall or other climate patterns, poverty and malnutrition, each contributing to emerging or re-emerging outbreaks of VL [5, 9, 11, 16, 17]. The VL transmission cycle caused by *L. donovani* in East Africa is generally considered to be anthroponotic (AVL) [18]. The AVL reservoir is comprised of humans with active infection from VL or post-kala-azar dermal leishmaniasis. However, some reports have suggested that a second zoonotic VL (ZVL) cycle is also present in some areas, in which dogs or rodents are also major animal reservoirs [15, 19]. ZVL caused by either *Leishmania infantum* or *Leishmania archibaldi* has also been observed in East Africa, such as the village of Barbar El Fugara in Al Qadarif, eastern Sudan, on the border with Ethiopia [15] (Table 1).

**Table 1:** Visceral leishmaniasis in East Africa

VL cycle	Causative agent, vector and disease reservoir	References
Anthroponotic (VL)	Parasite: *Leishmania donovani*	[11, 15, 17, 20, 22, 23, 45, 57]
	Vectors: *Phlebotomus* (*Larroussius*) *orientalis*; *Ph.* (*Synphlebotomus*) *martini*; *Ph.* (*Anaphlebotomus*) *rodhaini* ^a^; *Ph.* (*Synphlebotomus*) *celiae*	
	Disease reservoirs: Humans	
Zoonotic (VL)	Parasites: *Leishmania donovani*, *Leishmania infantum*, *Leishmania archibaldi*	[15, 22, 33, 57]
	Vectors: *Phlebotomus rodhaini* is possible vector for ZVL in East Africa	
	Disease reservoirs: Rodents (Egyptian mongoose) and dogs are possible reservoirs for ZVL cycle	

^a^ *Phlebotomus rodhaini* has been found to be infected with *L. donovani* in Eastern Sudan and is reported to be a possible vector for transmission of the disease between animal reservoirs

*Phlebotomus orientalis* is widespread throughout East African countries and is reported as the major vector for both AVL and ZVL in East Africa [11, 12, 18, 20]. *Phlebotomus orientalis* is correlated with dry seasons and with the presence of *Acacia seyal* forests, which are high abundant vegetation in Sudan and the lowland areas of Ethiopia [21]. In contrast, *Ph. martini* is reported in Uganda, Kenya, Somalia, southern regions of South Sudan and Ethiopia, where high moisture and relatively high humidity combine with moderate temperatures [12]. *Phlebotomus rodhaini* has been found to be infected with *L. donovani* in Eastern Sudan and is reported to be a possible vector for transmission of the disease between animal reservoirs [22, 23].

## Methods

Electronic searches were performed using PubMed and Google Scholar. Searches were limited to the years between January 1955 and January 2016. The searches consisted of three terms used in combination using the format “Term 1” AND “Term 2” AND “Term 3”. Term 1 included: “visceral leishmaniasis”, “post-kala-azar dermal leishmaniasis”, “cutaneous leishmaniasis” and “phlebotomine sand fly”. Term 2 included: “conflict”, “epidemiology”, “control”, “treatment” and “diagnostic”. Term 3 included: “South Sudan”, “Sudan”, “Ethiopia”, “Uganda” and “Somalia”. Articles included in this review stated clear and obvious methodology and results using molecular methodology for identification of parasites and morphological or molecular findings for identification of the phlebotomine sand flies. In addition to PubMed and Google Scholar, VL statistics in East Africa were also gathered from the World Health Organization (WHO) and the Software ArcGIS 10 (ESRI, Redlands CA) was used to map the distribution of VL cases across the East African countries.

### Epidemiology of VL in East African countries

**Fig. 1:**
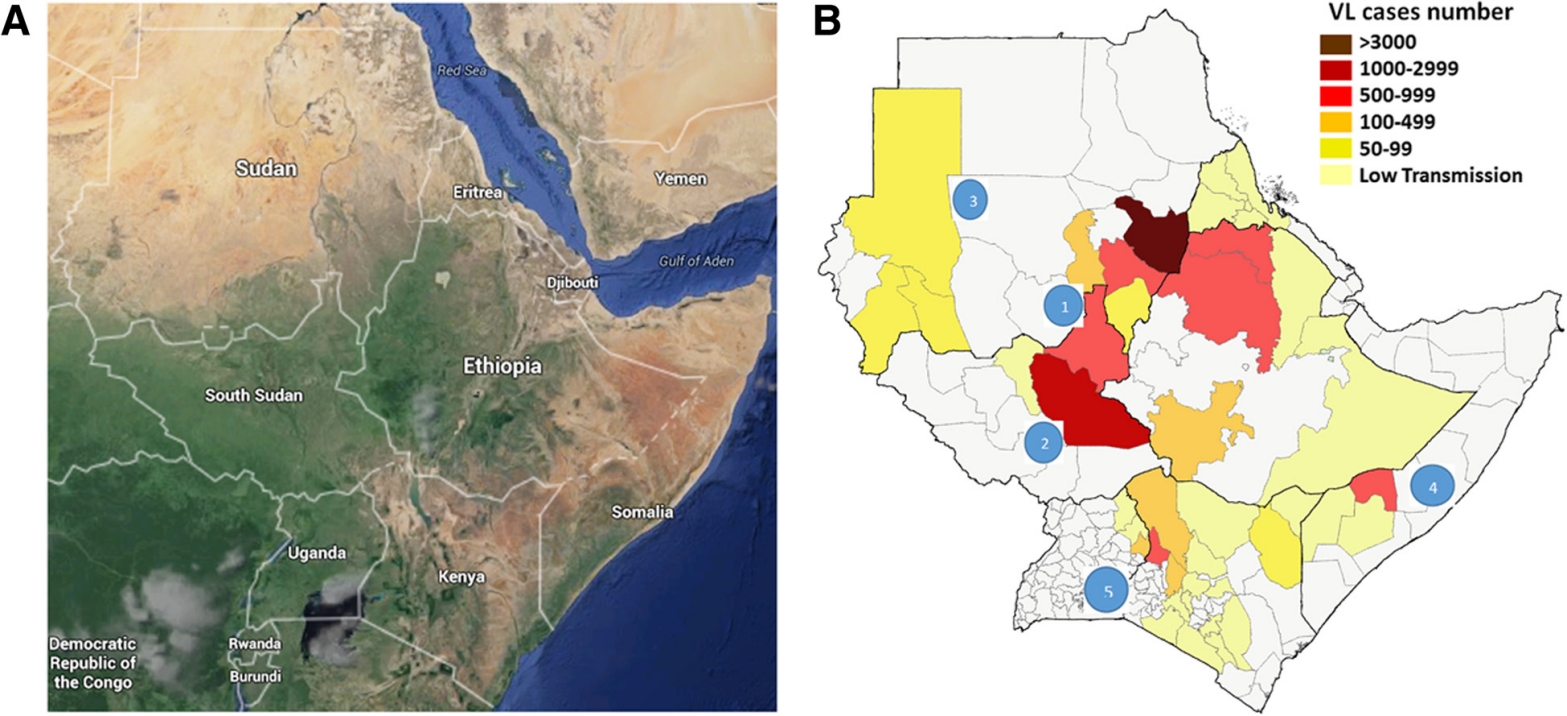
Distribution of visceral leishmaniasis (VL) cases in East Africa. **a** This satellite image is taken from Google Maps. **b** The distribution of VL for each region or state within East African countries [5, 27, 35, 36]. The largest affected area in terms of number of cases is the eastern region of Sudan and neighboring Ethiopia (Area 1), followed by South Sudan (Area 2), Darfur and Western Sudan (Area 3), and Somalia (Area 4), and Kenya with North East Uganda (Area 5)

VL is known to be highly distributed in East Africa, particularly in Kenya, Uganda, Sudan, South Sudan, Somalia and Ethiopia [11, 16]. Beyond the numbers highlighted in Fig. 1 additional estimates indicate 30,000 cases are reported annually in the region, with most cases probably caused by *L. donovani* (for most cases the etiologic agent is not routinely determined through molecular identification techniques, except for those cases specifically mentioned in the subsections below) [5, 24]. However, during times of conflict and in some post-conflict settings VL emerges in large and catastrophic human outbreaks. As shown in Fig. 1, East Africa’s VL cases concentrate in the eastern area of Sudan (where the largest number of cases are found) and across the border with Ethiopia. According to Alvar [5] the estimated annual VL incidence in Sudan is 15,700 to 30,300 [5]. Another important area is in the northeast region of South Sudan where the second largest numbers of cases are found with approximately 7,400 to 14,200 VL cases estimated annually along its border with Sudan and Ethiopia [5]. Additional foci are found in Ethiopia with an estimated 3,700 to 7,400 VL cases annually [5]. Annual estimates of VL incidence in Somalia are 1,400 to 2,700. Additional foci are located on the Kenyan Ugandan border and include estimates from 960 to 1720 annual VL incident cases [5].

#### Sudan and Ethiopia

Sudan reports approximately two thirds of the VL cases every year in East African countries, with most of them presumptively caused by *L. donovani* [25, 26]. Key external factors promoting VL in Sudan and Ethiopia include the environment and natural vegetation, poverty and crowding, and conflict [5, 24]. The Sudanese states of Al Qadarif, Sennar, and Blue Nile on the border with Ethiopia are reported as having a high risk of VL transmission, in part due to the *Acacia* forests. VL infection is highly correlated with sleeping under *Acacia* trees at night or engaging in night time outdoor activities near *Acacia* forests [27]. In addition to the *Acacia*, in both Sudan and across the border into the Ethiopian agricultural lowland states of Tigray and Amhara, thousands of VL cases also occur where large families live in crowded and poor conditions, frequently with cracked walls or thatched roofs (conditions favourable for phlebotomine sand flies) [28–32]. Another major factor with a seven-fold increased risk is found among people sleeping near dogs as *L. donovani* has been found in dogs and wild mammals in Sudan and Ethiopia [29, 33, 34]. Overall, social and political forces, especially military activities and agricultural development projects, are major drivers of VL endemicity and new epidemics in both Sudan and Ethiopia [11]. In Sudan a general lack of political will combined with the expulsion of some key aid agencies in 2009, has created further challenges in controlling VL in the endemic states of Al Qadarif, Sennar, Blue Nile and White Nile on Sudan’s eastern border with Ethiopia [24]. These factors are also exacerbated by human movements and unrest, depleted health care infrastructures, and normal seasonal movements of farmers. Compounding the situation and increasing the risk of VL are current food shortages and malnutrition affecting millions of people in the region. Also within Sudan but in areas away from its eastern border similar factors promote the spread of the disease within the interior of the country, especially in Darfur and Western Upper Nile [5, 30, 31], and across its southern border into South Sudan [9, 21, 24]. Details of the epidemic in South Sudan are discussed below.

#### Kenya, Uganda and Somalia

Looking south toward the Rift Valley, major VL disease foci can be found along the border between Kenya and Uganda [35, 36]. VL has also been reported as an endemic disease in Turkana, West Pokot and Baringo in Kenya, [35, 36]. Furthermore, VL epidemics in Kenya have been reported in Rift Valley and eastern regions, specifically Mandera and Marsabit [37, 38]. The Karamoja Region (specifically Nakapiripirit) in Uganda, one of the poorest regions of Uganda, is highly endemic for VL. In Somalia, the civil war has promoted the emergence of VL within the southern states of Bakool, Gedo and Bay [17, 37, 39]. Bakool in South Somalia is particularly known as a highly endemic region for VL and has experienced several disease outbreaks [31, 39]. In addition, a re-emergence of VL occurred during the Somalian civil war in Bakool, Gedo and Bay [17, 39] and has been reported among Somalian refugees in Kenya. VL human infections in Kenya, Uganda, and Somalia have been identified through molecular techniques *L. donovani* while a few have been diagnosed as *L. infantum* in Somalia [38, 40–42].

### The special case of South Sudan: impact of civil war, unrest, human displacement and refugee camps

**Fig. 2:**
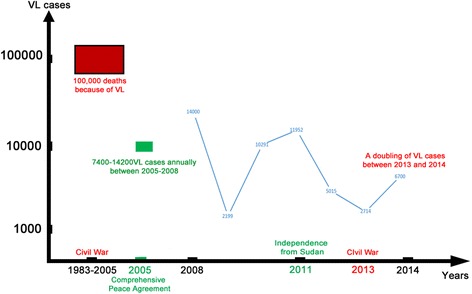
Timeline of political unrest and number of VL deaths and cases in South Sudan [15, 38–41]. Between 1983 and 2005 VL claimed approximately 100,000 lives in Southern Sudan. During this time there was also an on-going civil war in Sudan. In 2005 a Comprehensive Peace Agreement was signed. Between 2005 and 2008 an estimated 7,400 to 14,200 VL cases were reported annually in South Sudan. In 2011, South Sudan gained independence from Sudan and VL cases declined sharply to fewer than 5,000 cases in 2012 and to the lowest level in 2013 when only 2,714 cases were reported in South Sudan. In 2013 the nation of South Sudan entered into a civil war and experienced a doubling of the number of cases of VL cases in Lankien, Jonglei State and Malakal in Upper Nile State in 2014 with more than 6,700 cases treated. The exact number of cases between 1983 and 2005 is unknown, but must have been well over 100,000

South Sudan exhibits one of the world’s highest concentrations of NTDs and malaria. In addition to VL, major NTDs include human African trypanosomiasis, onchocerciasis, dracunculiasis, trachoma, onchocerciasis, Buruli ulcer, leprosy and intestinal helminth infections [43]. The long history of civil war in South Sudan has promoted major epidemics producing thousands of VL cases as shown in Fig. 2. Briefly, three distinct political eras have occurred since the civil war broke out and extending until the declaration of independence of southern Sudan from Sudan. The first era began in 1983 when a civil war started between Southern Sudan and Sudan, continuing until 2005 when South Sudan signed a Comprehensive Peace Agreement with Khartoum [44]. The 22 years of civil war between 1983 and 2005 resulted in the forced displacement of more than 4 million people, as well as one of the world’s worst lethal outbreaks of VL, claiming approximately 100,000 lives [18, 44]. The second era began in 2005, when due to the Comprehensive Peace Agreement [44] Khartoum could no longer offer practical support to South Sudan for the control of communicable diseases, including VL [43]. As a result, an estimated 7,400 to 14,200 VL cases were reported annually, particularly in Upper Nile, Unity, Jonglei and Eastern Equatoria, prior to the formal declaration of independence in 2011 [43, 45]. Subsequently, a third era began in 2011, with the South Sudanese Government allocating resources to overcome communicable diseases including VL. With tensions reduced in South Sudan, VL cases declined sharply to fewer than 5,000 cases in 2012 and to the lowest level in 2013 when only 2,714 cases were reported [45, 46]. Serological diagnoses using the rK39 rapid immunochromatographic and direct agglutination tests have been applied to diagnose VL cases in South Sudan [45], however, there is a lack of information regarding specific etiologic agents identified through molecular methods.

Unfortunately, this period has now ended. The most recent South Sudanese civil war between groups led by opposition leader Riek Machar and the South Sudan military led by President Salva Kiir Mayardit has forcibly displaced more than two million people either internally or to neighbouring East African countries since 2013 (Fig. 2) [47]. One estimate suggests that there have been over 1.5 million displaced refugees, of which 90 % have migrated to South Sudan states known to be highly endemic for VL, including Jonglei, Upper Nile, Unity and Eastern Equatoria [48]. More than 800,000 have fled to neighbouring countries [48]. The United Nations Office for the Coordination of Humanitarian Affairs (UNOCHA) and Médecins Sans Frontières (MSF) have identified malaria and other NTD outbreaks in areas affected by the civil war and unrest, with VL the most serious and lethal [18, 45]. MSF recently reported a doubling of the number of cases of VL cases in Lankien, Jonglei State and Malakal in Upper Nile State in 2014 with more than 6,700 cases treated compared to just 2,714 cases reported in 2013. With continued conflict, and resulting human displacement and lack of access to healthcare or facilities, the situation is expected to worsen [49], and then spread to neighbouring countries.

**Fig. 3:**
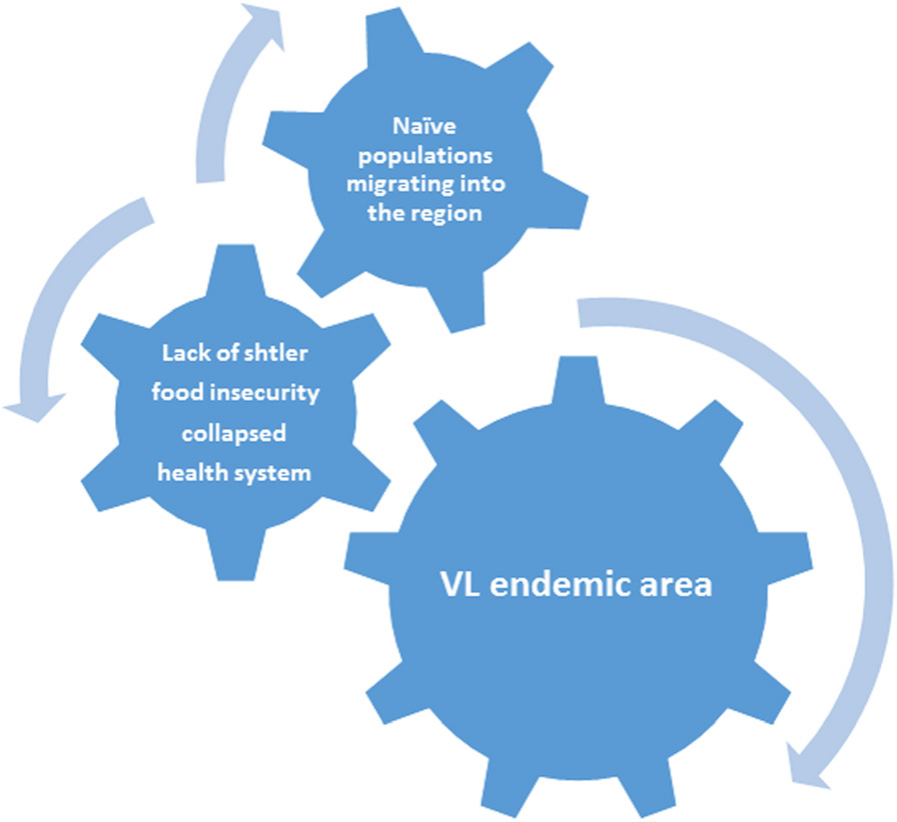
The “perfect storm” of conditions leading to VL Epidemics [35, 45–47]. The overarching problem in South Sudan since 2013 has two major factors. The first is a large population of naïve individuals migrating to endemic regions. In this case, migrations usually occur due to civil unrest leading to the second factor: a breakdown in infrastructure. This breakdown has led to lack of adequate shelter, food insecurity and collapsed healthcare systems, which leave individuals highly vulnerable to exposure to phlebotomine sand flies

The overarching problem in South Sudan since 2013 has four major components (Fig. 3). First those living in endemic conflict areas lack access to shelter and healthcare and are therefore highly vulnerable to exposure to phlebotomine sand flies and VL. Second, in some cases the displaced populations are immunologically naïve populations migrating into highly VL endemic regions [37, 50, 51], or conversely South Sudanese refugees fleeing VL-endemic areas are establishing new VL-endemic foci in neighboring countries. Finally, malnutrition resulting from food insecurity is an ongoing risk factor that is highly correlated with VL [52], and currently, deadly food shortages are affecting nearly 4 million South Sudanese according to UNOCHA. This current level of food insecurity is exacerbated by the current conflict.

**Fig. 4:**
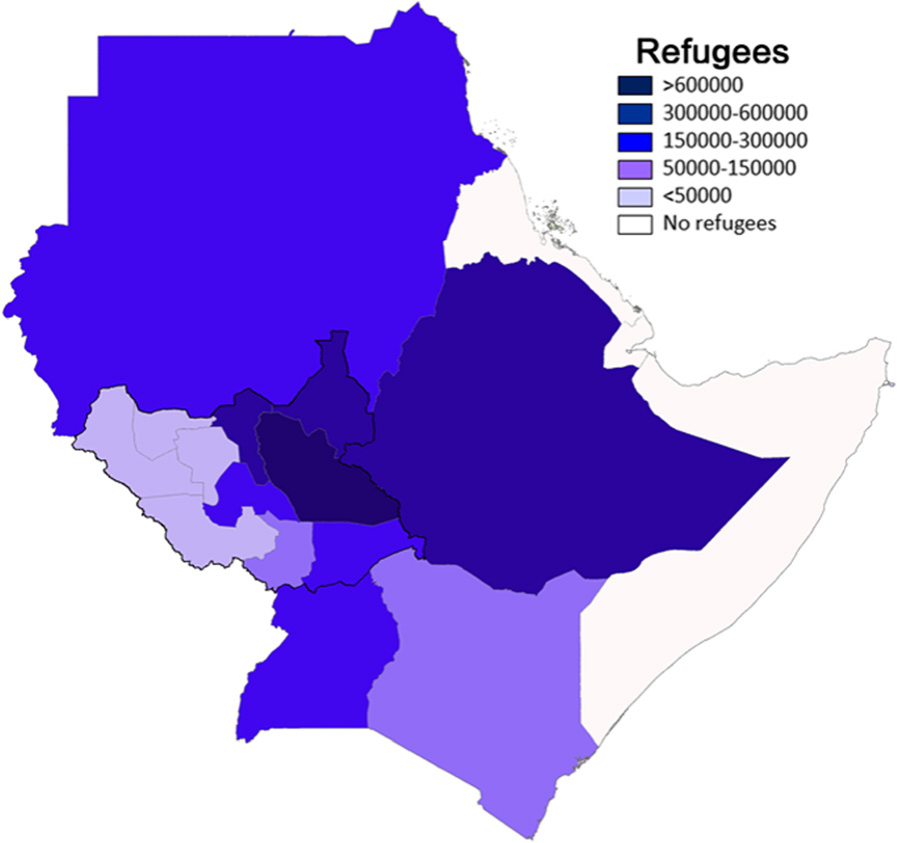
Number of refugees displaced internally or to neighbouring East African countries between 2014 and 2015 [28–30, 34, 36, 42, 43, 48–55]. The number of refugees in East African nations of Sudan, Eritrea, Djibouti, Somalia, Ethiopia, Kenya, Uganda and the states of South Sudan are shown. Numbers range from 0 to less than 50,000 to over 600,000 and are represented by intensity of color on the map

Figure 4 emphasizes the particular concern arising from the second factor highlighted above, namely the poignant human tragedy of South Sudanese refugees fleeing the conflict to neighbouring countries where VL is already endemic or beginning to emerge, or the potential for VL-infected refugees to create new endemic foci. Briefly, approximately 100,000 South Sudanese refugees fled toward the Northern Kenyan States and Rift Valley Regions, which are also known to be main foci for VL in Kenya [36, 38, 53–55]. Sudan has received approximately 200,000 South Sudanese refugees despite its own economic crisis and civil unrest [56]. More than one-half of the South Sudanese migrants that have fled to Sudan settled in the woodland region of White Nile state, which has reported high levels VL transmission and outbreaks [57–59]. In Ethiopia, South Sudanese migrants have fled to refugee camps in the southern communities of Omo Valley and Gambela, which are also known to be endemic foci for VL. Approximately 20 % of the total VL cases in Ethiopia, are reported to come from areas that host more than 300,000 South Sudanese refugees [30–32, 60].

**Fig. 5:**
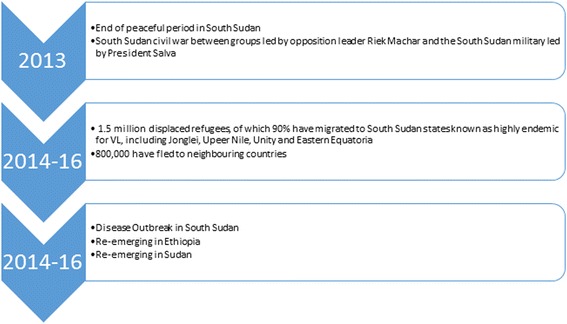
Consequences of conflict on South Sudan and neighbouring countries [44, 56]. This timeline represents the human toll of the recent political unrest in South Sudan and neighbouring countries. Consequences include not only forced displacement, but also outbreaks of VL due to the factors listed in Fig. 3

Figure 5 shows a summary of these recent events in South Sudan, which includes the rise of internal opposition and forced internal displacement of 1.5 million people and heightened vulnerability to VL. The external displacement of 800,000 people to neighbouring countries is helping to establish new VL foci or re-emerging outbreaks of VL. There is a dearth of published information about these events. For instance, anecdotal reports state VL is emerging among South Sudanese in Ethiopia [49, 61]. Kenya remains without any written reports from Kakuma, which is endemic with VL.

### Other aspects of VL in East Africa: The added challenges of post-kala-azar dermal leishmaniasis (PKDL) and complicating host factors

A major complication of VL is PKDL, which is characterized as a macular, papular, or nodular rash that often begins at the mouth, and is seen with especially high frequency in East Africa where it can occur in up to 50 % of treated patients [18, 62, 63]. On the Indian subcontinent, 400 specimens of laboratory-bred *Ph. argentipes* have been diagnosed via xenodiagnosis. A quarter of phlebotomine sand flies fed on PKDL nodular cases, and a half of surviving phlebotomine sand flies became infected with the *Leishmania* parasite [63, 64]. Furthermore, an observational study was carried out in Sudan on an infected boy with nodular PKDL resulting in 25 % infection of *Ph. orientalis* that fed on the nodules [63, 65]. Thus, PKDL patients can act as reservoir for VL infection [66, 67]. As a result, *Ph. orientalis* sand flies are able to transmit VL infection from PKDL patients, especially those in refugee camps where there is increased exposure to the phlebotomine sand fly [68, 69].

A recent study conducted in India suggested a strong association between testosterone and PKDL in India [70]. However, in East African countries PKDL patients developed disease after receiving inadequate and irregular antimonial treatment of kala-azar [14, 70, 71]. Moreover, it is hypothesized that PKDL patient lesions are linked to sun-exposed areas on the body in East African countries [72]. UV light is hypothesized to play a role in parasite persistence and is reviewed elsewhere [71, 72]. Mukhopadhyay et al. [71] state that PKDL could develop as a result of reinfection or parasite persistence and also could be explained by a failure of tissue-specific T cell memory or as a consequence of genetic background susceptibility.

Treatment choices for PKDL are pentavalent antimonials or liposomal amphotericin B that requires cold storage [9, 24]. In areas experiencing extreme conflict, treatments requiring cold storage are unrealistic. Therefore, pentavalent antimonials that have been licensed in East African countries are the most common choice [24]. Patient treatment for both VL and PKDL is further complicated by the absence of reliable field-based diagnostics. For example, the rK39 rapid diagnostic tests that is currently recommended by the WHO for use in primary health care centres has a lower sensitivity to VL among East African populations relative to other populations possibly due to chronic malnutrition with zinc deficiency among VL and PKDL patients in East African countries [9, 73, 74]. Other diagnostic tools such as direct agglutination test, microscopy on bone marrow, lymph node or spleen aspiration require well-equipped facilities within the district hospital level which is difficult to achieve in areas under conflict [9, 18, 75–77]. Diagnostic and treatment tools and algorithms need to be revised.

Still other major challenges to VL in East Africa are high underlying levels of HIV/AIDS co-infection and malnutrition. Treatment failure rates are common in both populations. [78]. In North Ethiopia 40 % of VL patients are co-infected with HIV [79, 80]. Moreover, a serious level of malnutrition has been reported in most parts of South Sudan, particularly among children. Because malnutrition is highly correlated with VL, this situation may lead to additional outbreaks of VL in South Sudan in the future [17, 52, 81, 82].

### Preliminary public health and policy recommendations

#### Enhanced disease surveillance: an expanded program of enhanced disease surveillance is urgently needed for VL and other NTDs

Strengthening the surveillance system amongst displaced populations, such as refugee camps, will be crucial to treating current cases and avoiding re-emergence of VL. Surveillance systems can be applied actively through clinical investigation and diagnostic tools - although the diagnostic tools in East African countries are not always as highly sensitive as when they are used to diagnose populations from other regions [73]. The fact that PKDL affects more than half of individuals previously cured of VL is another concern for maintaining disease reservoirs. There is an urgent need to improve the diagnostic tools required to implement VL screening in East Africa, to ensure that they are suitable for field purposes [9, 18, 68, 69, 83, 84]. Since the rK39 immunochromatographic test has significantly lower sensitivity in East African countries, other diagnostic tools should be introduced with appropriate clinical investigations for sensitivity and specificity in this population. One option is the direct agglutination test (DAT), with careful consideration to the conflict situations and with proper supervision and quality assurance as described in WHO recommendations [9, 84–86]. Until a more reliable test is available for use in these populations, individuals in East Africa with clinical symptoms of VL and negative rK39 should have their cases confirmed with DAT whenever possible in order to accomplish basic active surveillance [9, 35, 74, 87].

#### Case detection and treatment: enhanced efforts are needed to increase access to treatment, while parallel efforts are needed to determine optimal treatment regimens

Beyond surveillance, access to treatment must be provided when cases are detected (Table 2). The toxicity of drug treatment, PKDL, malnutrition, HIV co-infection and collapsed health care facilities are all major issues facing health care workers providing treatment for VL in areas under conflict, such as refugee camps within South Sudan and in neighbouring countries. Pentavalent antimonials are licensed in all East African countries and are the standard first-line medicines for VL [9, 24]. However, adequate treatment with antimonial drugs requires better access to health care services in these areas [9, 24, 88]. An alternative choice is amphotericin B deoxycholate and pentamidine [9]. For VL cases with HIV co-infection it has been reported that miltefosine in combination with liposomal amphotericin B, followed by secondary prophylaxis and antiretroviral drugs gives promising results in treatment response [78]. For cases of PKDL WHO recommends that pentavalent antimonials or liposomal amphotericin B should be used in East African countries [9]. However, increasing resistance to antimony treatment in more than half of VL patients has been reported in some areas in East Africa [24]. The oral drug, miltefosine, has shown promising results to treat PKDL and may be considered as an alternative as resistance to antimonials concerns are rising [83].

**Table 2:** Treatment recommendations for VL in East Africa

Treatment phase	Recommended therapy	References
First line VL	Pentavalent antimonials	[9, 24]
Second line VL	Amphotericin B deoxycholate and pentamidine	[9]
First line VL and HIV Co-infection	Miltefosine + liposomal amphotericin B + antiretroviral therapy	[68]
First line PKDL	Pentavalent antimonials, miltefosine or liposomal amphotericin B	[9, 24, 59, 81]

#### Vector control: integrated vector control measures must be implemented in addition to evaluation of new or existing technologies, including insecticide-treated bed nets

Traditional methods of vector control work. It is important to fill gaps and cracks in building walls with lime plaster, mud or cement as living in the houses with cracked walls increases the possibility of contracting VL infection as consequences of increased exposure to phlebotomine sand fly bites [28]. A case control study carried out in north-western Ethiopia showed both cracked house walls and cracked black soil near houses boosted risk of transmission with an odd ratio of 2.768 and 6.266, respectively [28]. Additionally, use of bed nets and also the traditional habit of incense-burning plants will reduce bite exposures inside houses [28]. Impregnated materials, such as insecticide-treated bed nets (ITNs) and curtains offer alternative solutions to reduce the abundance and activity of phlebotomine sand flies inside and around residential houses [89, 90]. Two thirds of kala-azar cases have been reduced after insecticide impregnation bed-nets applied in Bangladesh [89]. Lambda-cyhalothrin impregnated bed nets were supplied to two villages in Dinder National Park, Sudan and were found to be protective, reducing VL cases by 59 % [91]. Furthermore, Ritmeijer et al. [92] suggest that the efficiency of ITNs is depends on behavioural factors, which vary among different communities. Fine-mesh ITNs were very effective when introduced in highly endemic areas in Eastern Sudan using pyrethroid, with an estimated 60 % reduction of VL cases after ITN intervention [92]. Additionally, repellent gives high protection from *Ph. orientalis* as described by MSF-Holland [79].

#### Multilateral initiatives: several multilateral initiatives related to eliminating VL and other NTDs in South Sudan and other East African countries are underway and require unprecedented commitments. Cessation of current hostilities would enhance the success of these programs

Today, VL is an integral component of the ongoing humanitarian tragedy in East Africa. The most recent World Health Assembly (WHA) resolution specifically directed at intervening against VL is WHA 60.13 adopted in 2007. In 2013, WHA66 re-emphasized the importance of the global control, elimination and eradication of all NTDs. At the end of 2014 several African countries, including Sudan and South Sudan, signed the Addis Ababa NTD Commitment, in which each country pledged to enhance efforts to ensure that WHO goals for NTD control, elimination and eradication by 2020 are met. The commitment includes guaranteeing the implementation of NTD programs that contribute to strengthening the overall health system, increasing domestic contribution to NTD programs, improving national coordination of NTD program goals, and timely reporting and use of NTD program data. In line with these goals, South Sudan recently launched a master plan to eliminate NTDs over the next five years from 2016–2020. Success of this plan will rely heavily on international cooperation and a de-escalation of the current civil upheaval.

In 2011 WHO brokered a deal with Gilead, the manufacturer of AmBisome (liposomal amphotericin B), for the donation of 445,000 vials to countries including Ethiopia, Sudan and South Sudan, to treat over 50,000 VL patients through 2017. While this was a great contribution to the fight against VL, its impact will be modest, as there are currently hundreds of thousands of cases of VL and millions of people at risk. MSF is calling for a reduction in the price of liposomal amphotericin B to accelerate its rollout to more patients.

Other international commitments to fight NTDs in South Sudan are also currently underway. The United States Agency for International Development works with RTI International, the government of South Sudan and Ministry of Health, and a wide range of stakeholders to implement an NTD program in the region since 2008. In a recent partnership the Drugs for Neglected Diseases *initiative* (DND*i*), the British Department for International Development, the London School of Hygiene and Tropical Medicine, Mott McDonald and MSF launched the Consortium for the Control and Elimination of VL, known as KalaCORE, which focuses its efforts in high-prevalence countries in South Asia and East Africa. This new effort complements the Leishmaniasis East Africa Platform (LEAP) founded by DND*i* in 2003 in collaboration with international stakeholders to strengthen capacity against leishmaniasis in the region.

#### Strengthened health systems

South Sudan’s current civil war crisis has destroyed its health care infrastructure. There is an eerie resemblance to the collapse that preceded Ebola outbreaks in Guinea, Liberia and Sierra Leone. Attacks have destroyed hospitals that for decades have provided care for VL, HIV/AIDS, tuberculosis, malnutrition and other diseases and conditions. Such attacks are not new, and are in clear violation of International Humanitarian Law. On December 11, 2015, MSF called for an increased humanitarian response to the current conditions in South Sudan, including relief of malnutrition and increased access to health care. This statement echoed the sentiments expressed by the Secretary General of MSF, Jérôme Oberreit, the year before, when he also called for parties to the conflict to ensure that all people in South Sudan are able to seek health care without fear of violence.

Other important issues related to VL infection that must be addressed are malnutrition and the cost of treatment. Currently, the UN World Food Program is severely underfunded, making it difficult to provide the nutritional assistance that is necessary in South Sudan and other areas affected by hunger. In terms of treatment cost, a 2013 study of the economic cost of VL in Sudan (the only one of its kind so far for East Africa) showed significantly high costs for both health care providers and affected households [93]. This high economic burden was found to be mostly associated with the high cost of the pentavalent antimonial used as first-line treatment and the long treatment course of 30 days required for effective treatment [93]. Food costs during treatment were also found to be an important contributor to the high cost of treatment for households [93]. Utilizing alternative treatment strategies, such as those mentioned above, which can reduce total treatment time, may help reduce the economic burden until a more cost effective solution, such as a vaccine against VL, is available.

Recently, the United Nations included a target to end NTDs by 2030 in the Sustainable Development Goals, a clear global acknowledgement of the effect that NTDs have on overall human development. Now it is up to individual countries and international aid organizations, both governmental and non-governmental to fully commit to this goal. For VL in South Sudan and the rest of East Africa, in addition to research, disease surveillance and vector control, achieving this goal will involve national and international political will to end the on-going violence, relieve current food insecurity, and strengthen the overall health care system in the region.

## Conclusions

We find a strong relationship between civil unrest and VL epidemics which tend to occur among immunologically naïve migrants entering VL-endemic areas and when *Leishmania*-infected individuals migrate to new areas and establish additional foci of disease. Further complicating factors in East Africa’s VL epidemics include severe lack of access to diagnosis and treatment, HIV/AIDS co-infection, food insecurity and malnutrition. Moreover, cases of post-kala-azar dermal leishmaniasis (PKDL) can serve as important reservoirs of anthroponotic *Leishmania* parasites.

## Abbreviations

DALYs: Disability-adjusted life years
DAT: Direct agglutination test
ITNs: Insecticide-treated bed nets
LEAP: Leishmaniasis East Africa platform
MSF: Médecins Sans Frontières
NTDs: Neglected tropical diseases
PKDL: Post-kala azar dermal leishmaniasis
VL: Visceral leishmaniasis
WHA: World Health Assembly
WHO: World Health Organization
UNOCHA: United Nations Office for the Coordination of Humanitarian Affairs
